# Evidence for independent evolution of functional progesterone withdrawal in primates and guinea pigs

**DOI:** 10.1093/emph/eot022

**Published:** 2013-12-03

**Authors:** Mauris C. Nnamani, Silvia Plaza, Roberto Romero, Günter P. Wagner

**Affiliations:** ^1^Yale Systems Biology Institute and Department of Ecology and Evolutionary Biology, Yale University, New Haven, CT, USA; ^2^Perinatology Research Branch, Program for Perinatal Research and Obstetrics, Division of Intramural Research, Eunice Kennedy Shriver National Institute of Child Health and Human Development, NIH, Bethesda, MD, USA; ^3^Department of Obstetrics and Gynecology, University of Michigan, Ann Arbor, MI, USA; ^4^Department of Epidemiology and Biostatistics, Michigan State University, East Lansing, MI, USA; ^5^Department of Obstetrics and Gynecology, Wayne State University, Detroit, MI, USA

**Keywords:** guinea pig cervix, gene expression, functional progesterone withdrawal, evolution of parturition

## Abstract

**Background and objectives**: Cervix remodeling (CRM) is a critical process in preparation for parturition. Early cervix shortening is a powerful clinical predictor of preterm birth, and thus understanding how CRM is regulated is important for the prevention of prematurity. Humans and other primates differ from most other mammals by the maintenance of high levels of systemic progesterone concentrations. Humans have been hypothesized to perform functional progesterone withdrawal (FPW). Guinea pigs are similar to humans in maintaining high-progesterone concentrations through parturition, thus making them a prime model for studying CRM. Here, we analyze the phylogenetic history of FPW and document gene expression in the guinea pig uterine cervix.

**Methodology**: Data on progesterone withdrawal were collected from the literature, and character evolution was analyzed. Uterine cervix samples were collected from non-pregnant, mid-pregnant and late pregnant guinea pigs. RNA was extracted and sequenced. Relative transcript levels were estimated and compared among sample groups.

**Results**: The phylogenetic analysis shows that FPW evolved independently in primates and guinea pigs. The transcriptome data confirms that guinea pigs down-regulate progesterone receptor toward parturition, in contrast to humans. Some of the similarities between human and guinea pig are: down-regulation of estrogen receptor, up-regulation of VCAN and IGFBP4 as well as likely involvement of prostaglandins.

**Conclusions and implications**: (i) FPW in guinea pigs evolved independently from that in primates. (ii) A small set of conserved gene regulatory changes has been detected.

## Abstract (precis)

Humans and guinea pigs differ from other mammals by maintaining high progesterone levels in pregnancy all the way through birth. Here we investigated the evolutionary history of this condition and conclude that it evolved independently in the human and the guinea pig lineages. Furthermore we investigated the gene expression during cervical re-modelling and found only a small number of gene regulatory events that seem to be common between humans and guinea pigs.

## INTRODUCTION

Cervical remodeling (CRM) is a necessary step in preparation for successful parturition [1]. Premature softening and shorting of the cervix is also a powerful predictor of preterm birth, and thus understanding the molecular mechanisms regulating CRM are critical for successful intervention to prevent prematurity [2]. A major obstacle in unraveling the mechanisms of human cervical ripening is that mammals are highly variable with respect to the mechanisms underlying parturition. Most notable is the fact that in humans and other primates the placenta produces large amounts of progesterone through pregnancy and labor. In contrast, most other placental mammals, including model organisms such as sheep, mouse, rabbit and rat, have systemic progesterone withdrawal, meaning that the serum concentration of progesterone declines toward term before the onset of labor. Declining progesterone concentrations are thought to remove the ‘progesterone block’ from the uterus and the cervix and thus allow progression toward parturition [3–5]. To explain parturition in humans, it has been suggested that there have to be changes downstream of the progesterone signal that undercut the progesterone block in its target tissues. Several mechanisms have been proposed for this so-called functional progesterone withdrawal (FPW) [6–9], but none of them have yet received decisive support.

A notable exception among model organisms is the guinea pig that, like the human, maintains high concentrations of serum progesterone through parturition [10]. It has thus been suggested that guinea pig is a potential model organism to study the mechanisms of FPW as well as prematurity, which is very rare in model species with fast gestation like mice [11]. This is an attractive possibility, as guinea pigs belong to the same clade as other major model organisms, mice, rat and rabbit, the so-called Glires [12–15]. It is also a model organism with a long tradition of experimental research [16] and a sequenced genome (http://useast.ensembl.org/Cavia_porcellus/Info/Index).

In this communication we investigate gene expression in the cervix of guinea pigs to assess the similarity between human and guinea pig CRM. Our aim is to answer the question of whether FPW in primates and guinea pigs is homologous, meaning that it was already present in the most recent common ancestor of humans and guinea pig. This question is important because molecular mechanisms are more likely shared among homologous characters than among characters that evolved independently. Guinea pig belongs to a basal rodent lineage [14], and it is thus possible that FPW in primates and guinea pigs could be homologous.

Here, we report that there are extensive differences between the human and guinea pig cervical gene expression dynamics, which makes it unlikely that the mechanisms of ‘FPW’ are homologous between the two species. This inference is also supported by a phylogenetic analysis of serum progesterone concentrations at parturition among Archontolgires, the clade uniting primates and rodents. Nevertheless, it is still possible that there are conserved molecular mechanisms shared between primates and other placental mammals, as for instance the down-regulation of estrogen receptor alpha (ESR1), as reported in our previous paper for humans [17] and here for guinea pigs.

## METHODOLOGY

### Tissue harvesting and RNA extraction

Hartley guinea pigs (pregnant and non-pregnant) were obtained from a commercial supplier (Charles River, Wilmington, MA, USA). The animals were housed in individual plastic cages with hay, branches and other environment enhancing objects. Non-pregnant females were examined daily for vulva occlusion membrane. These animals were sacrificed the day after the vulva occlusion disappeared indicating entry into estrus. Pregnant females were obtained with uncertain pregnancy dates and were euthanized based on assessing overall body weight of the female with gestation range provided by the suppliers. For ‘post-mortem’ assessment of the duration of pregnancy, we took measurements of both the fetal supra-occipital-premaxilla length and fetal weight at harvest (Supplementary Table S1). To estimate gestational age we used previously published data [18–20]. Estimated gestational stage for mid-pregnant and late pregnant (but not in labor) animals was 43.6 ± 0.5 (mean ± SD) days and 65.2 ± 1.2 days, respectively (average gestation period of 65.5 ± 6.5 days range [16]). Tissue harvesting focused on the vaginal portion of the cervix to minimize contamination with uterine myometrial tissue. Samples were collected from the three groups in triplicates for non-pregnant and mid-pregnant females and four replicates for late pregnant females. Cervical tissue was immediately stored in RNAlater Solution (Ambion, cat# AM7020) until further processing. Total RNA was extracted using the RNeasy extraction kit (Qiagen, cat# 75142) following manufactures instructions that included an in-column DNAse digestion. Quality of the RNA was then confirmed using Agilent 2100 Bioanalyzer (Santa Clara, CA, USA).

### Sequencing and data processing

RNA library preparation and high-throughput sequencing were performed on an Illumina HiSeq 2000 sequencing system following the protocol recommended by Illumina for sequencing total RNA samples. Sequencing was done for each biological replicates at 1 × 75 bp strand specific by the Yale Center for Genome Analysis. Sequence reads were aligned to the *Cavia porcellus* reference genome (cavPor3.69) using the splice junction mapper for RNA-seq reads TopHat2 [21–23]. Sequencing depth for RNA-seq samples averaged 45 million reads per biological sample with >80% overall alignment rate. After alignment, read counts were determined with HTSeq-count v0.5.4p1 as described by the authors. All RNA-seq data are deposited in GEO under accession number GSE47986.

### Data analysis

Relative RNA abundance was measured as transcripts per million (TPM) transcripts as recommended in [24, 25] rather than RPKM or FPKM. The reason is that units of RPKM are not consistent between samples and are thus problematic when comparing RNA abundance between samples of different transcript composition [25].

In comparing transcript abundances we distinguish two different kinds of events. At the one hand are induction and repression of gene expression (turning gene expression ON or OFF). On the other hand are modulations of gene expression. We refer to induction when the transcript abundance of a gene is below the operational threshold of three TPM [26, 27] in the earlier stage of the reproductive cycle but above the threshold in the later stage. The threshold of three TPM corresponds to ∼1 RPKM in terms of the traditional RNA abundance measure [26, 27]. The threshold is based on association between expression level and chromatin modification status as well as a statistical model of transcript abundance. In contrast, we call changes of transcript abundance ‘expression modulation’ if the estimated transcript abundance is above the operational threshold in both stages compared. When we refer to up or down regulation, we mean gene expression modulation.

The motivation for making the (operational) distinction between gene expression modulation and induction/repression is that they represent two different biological events. Induction/repression is associated with qualitative changes in the nature of the chromatin modification and the kind of transcription factors and co-factors associated with the *cis*-regulatory elements [27]. On the other hand, gene expression modulation can have a variety of causes, from phosphorylation status of transcription factors to the expression level of upstream regulators.

### Statistics

When correlations were calculated or differential expression was tested, we transformed TPM data by square root transformation rather than the more widely used log-transformation for two reasons. First, TPM as well as RPKM data usually contains zero values. In a log-transformation these data points will be transformed into minus infinity, with consequences for data handling and interpretation. In contrast, $\sqrt{0}=0$ and no further problems arise. The second reason to prefer square root transformation is that it is in fact variance stabilizing [26], while the log-transformation leads to an inflation of variance at low abundance values. Tests for differential expression were not used as a discovery tool but to test specific hypotheses. For instant, we tested differences between stages for those genes that have been found differentially expressed in humans. For this reason, we did not use multiple comparison corrections. We used one-way ANOVA and *t*-tests on square root transformed TPM data.

### BrdU labeling

Three guinea pigs 35–38 weeks pregnant for mid-pregnant and also three females 58–63 weeks pregnant for late pregnancy were obtained from Charles River laboratories. Two females from each group were injected intraperitoneal with 10 ml of Bromodeoxyuridine (BrdU) labeling reagent Life Technology (Cat # 0103), and one control sample with 10 ml PBS. After 24 h the animal was sacrificed, and cervix was recovered and fixed in 4% paraformaldehyde (24–48 h). The tissue was processed for paraffin embedding and serial section of 6 µm was produced. The slides were processed following the Life Technology protocol for BrdU (Cat # 93-3943).

## RESULTS

### Evolution of functional progesterone withdrawal

The guinea pig has attracted interest because it shares with humans the feature of maintaining high concentrations of serum progesterone (P4) (>200 ng/ml) through parturition [10]. For both species it has been suggested that some mechanism downstream of the progesterone signal is responsible for CRM, called FPW. It is thus interesting to ask whether FPW in the guinea pig is homologous to that in humans.

**Figure 1.**
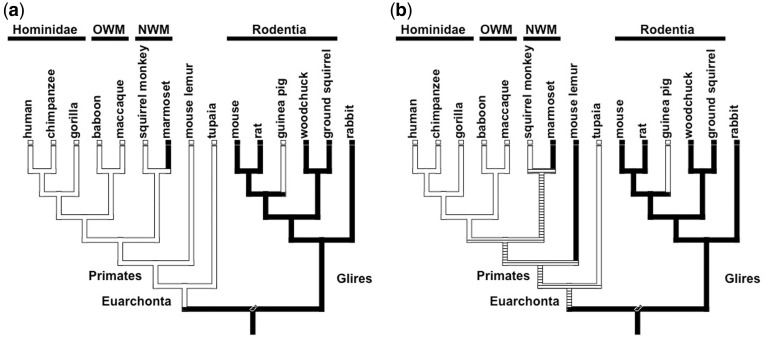
Evolution of FPW. Phylogenetic reconstruction of serum progesterone concentration at parturition in Euarchontoglires, the clade uniting primates and rodents. Species and lineages with sustained high systemic progesterone concentrations are indicated in white, species and lineages with a drop of systemic progesterone concentrations are indicated in black. The root state was fixed as progesterone withdrawal based on outgroup comparison with hoofed animals and carnivores. Tree topology in Glires follows fig. 6 in [28]. **(a)** Scenario that assumes that the mouse lemur has progesterone withdrawal. Under this assumption the lack of systemic progesterone withdrawal is an ancestral state of primates and even the Euarchonta. **(b)** Scenario that assumes that the mouse lemur does not have progesterone withdrawal. Under this scenario the ancestral state for Euarchonta and primates is ambiguous. Note that in both scenarios guinea pig is the only lineage without progesterone withdrawal within the Glires and that guinea pig is deeply nested within this clade. This phylogenetic distribution suggests that FPW evolved independently in primates (Euarchonta) and guinea pig within the Glires.

In Fig. 1a and b, a survey of progesterone withdrawal within Euarchontoglires is presented based on the phylogenetic hypothesis in [28]. Lack of systemic progesterone withdrawal is found in humans, apes (chimpanzee [29], gorilla [30, 31]) and old world monkeys (rhesus monkey [32, 33], baboon [34]). The situation in new world moneys is complicated. The detailed study by Chambers and Hearn on the common marmoset (*Callithrix jacchus*) shows a steady decline in serum progesterone levels in the 4 days leading up to parturition [35]. Other authors did not report such a drop but also did not explicitly document the peripartum period [36]. In contrast, Corousos and collaborators report the peripheral progesterone levels of two pregnant squirrel monkeys (*Saimiri sciureus*) but do not show a decline of progesterone levels in the days leading up to parturition [37], even though the levels at the day of parturition are reported to be close to zero. Nevertheless, it is clear that the withdrawal, if any, would have to be very precipitous or absent. Based on comparison with the rate of decline in the marmoset we concluded that the squirrel monkeys do not have systemic progesterone withdrawal. We could not find data about tarsiers and some limited information about lemurs. Blanco and Meyer report progesterone levels in various stages of estrus and pregnancy of the brown mouse lemur (*Microcebus rufus*) but the gestational stages in this study are very unreliable [38]. In spite of that, the pre-partum cohort shows higher progesterone than the pre-estrus cohort, making it possible that the mouse lemur lack systemic progesterone withdrawal. Thus, we provide two reconstructions one with and one without systemic progesterone withdrawal for the mouse lemur (Fig. 1a and b).

An outgroup clade of primates are the Tupaias (Scandentia). In *Tupaia belaneri*, there is a drop in serum P4 only after parturition [39] but this paper reports unpublished data from Elger and Hasan about a single animal. However, the peak progesterone level is reported to occur 2 days before parturition and we thus classify it as having no systemic progesterone withdrawal. Within the Glires, the clade including rodents and rabbits and squirrels, all lineages have progesterone withdrawal except guinea pigs [40, 41].

In Fig. 1a and b, we provide two parsimony-based ancestral character state reconstructions reflecting the ambiguity about the situation in the lemur. Under either scenario the lack of systemic progesterone withdrawal in the guinea pig is reconstructed as independently derived (Fig. 1a and b). The difference between the two scenarios, lemurs with or without progesterone withdrawal, only affects the reconstruction of the ancestral character state in the primate lineage (Euarchonta). Assuming that the lemur has no systemic progesterone withdrawal (Fig. 1a) leads to a reconstruction where this condition is ancestral for the Euarchonta, and the situation in the marmoset is inferred to be a reversal. Assuming that the lemur has systemic progesterone withdrawal (Fig. 1b) leads to a reconstruction where the ancestral state of the stem lineage of primates is ambiguous. From these data we conclude that FPW likely has evolved independently in primates and guinea pigs.

### Gene expression in guinea pig cervix

Cervical tissue was harvested from guinea pigs in three reproductive stages: non-pregnant and in estrus (NP), in mid-pregnancy (MT) and late pregnancy/term (LT) (see ‘Methodology’ for details on the timing of sampling). The samples were transcribed and sequences with 1 × 75 bp to an average sequencing depth of 40–50 Mio reads. Reads were mapped to the guinea pig genome version cavPor3.69 and read counts transformed to TPM transcripts [25]. Below we focus on two comparisons of transcript abundance. One is the differences between non-pregnant and mid-pregnant stages and the other the comparison of mid-pregnancy and late pregnancy.

### Gene expression changes in the cervix from estrus to mid-pregnancy

**Figure 2.**
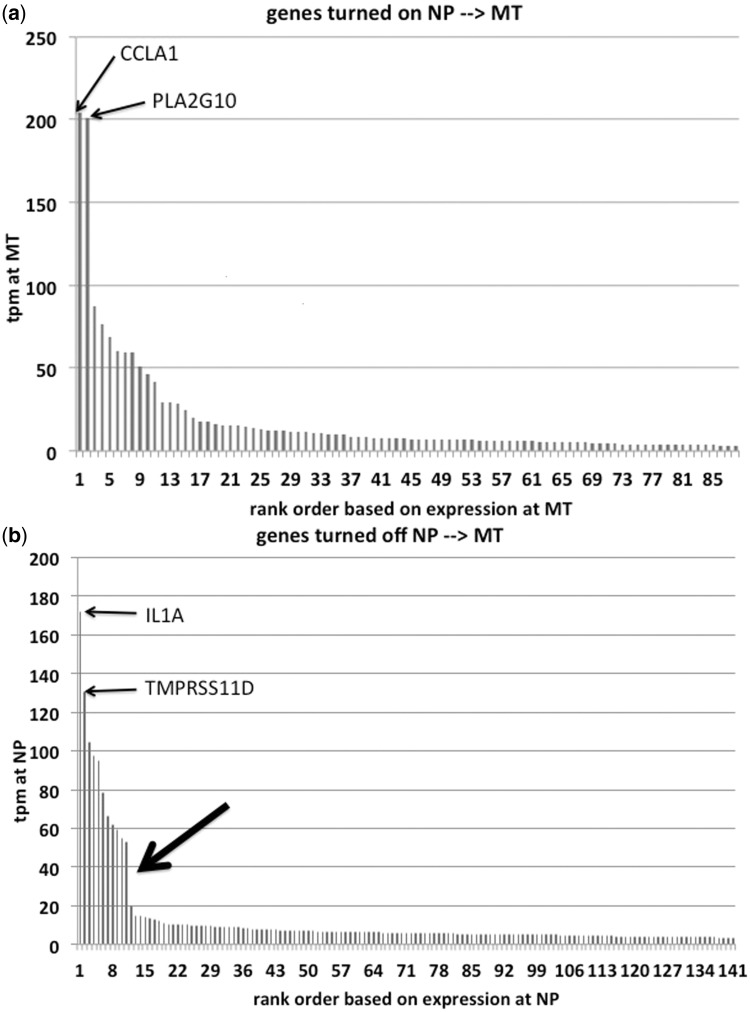

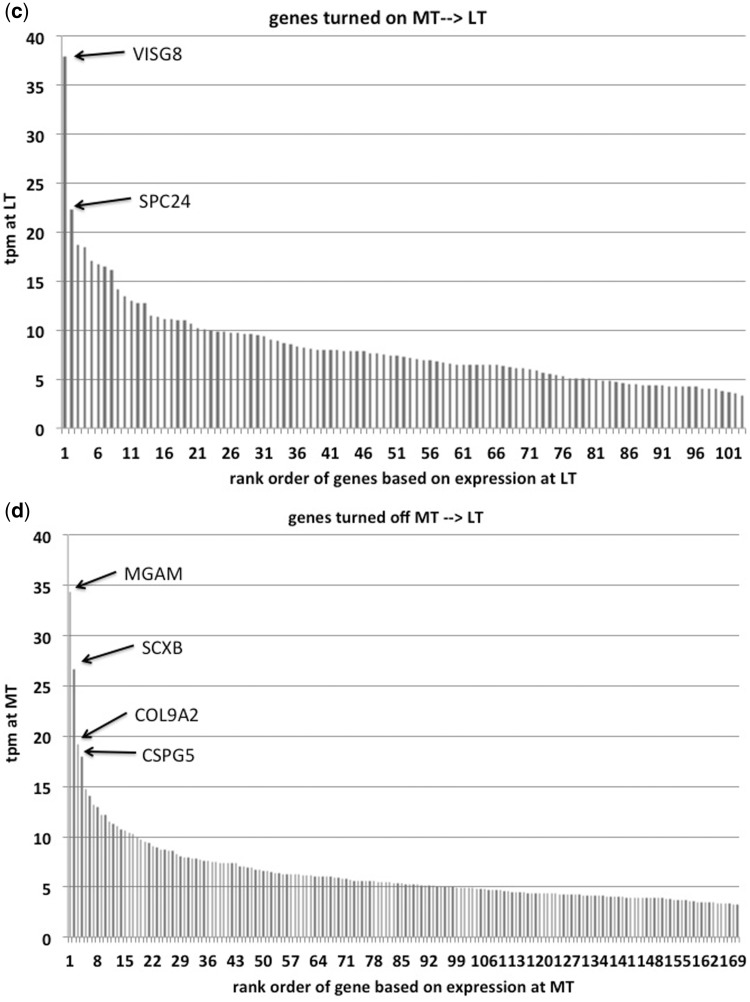

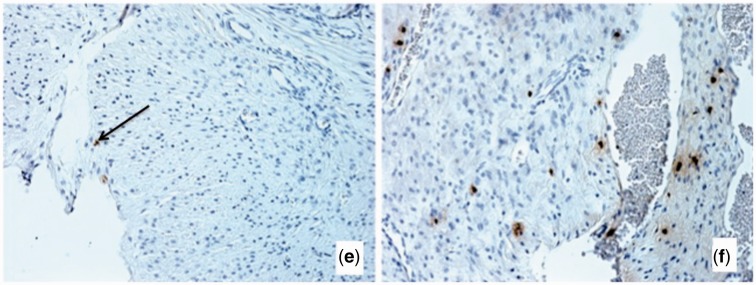
Expression levels in TPM of genes that are turned on or off during pregnancy in the guinea pig cervix. **(a)** Expression level of genes in mid-pregnancy of genes that are not expressed in non-pregnant cervix and expressed in mid-pregnancy. Note that there are two genes much more highly expressed than the others, CLCA1 and PLA2G10. **(b)** Expression level of genes in non-pregnant cervix that are not expressed in mid-pregnant cervix. There is a distinct difference in expression level between the 11 highest expressed genes and the rest (large arrow). **(c)** Expression level of genes in late pregnancy of genes not expressed in mid-pregnancy. **(d)** Expression level of genes during mid-pregnancy that are not expressed in late pregnancy. Four genes stand out: MGAM is the maltase-glucoamylase or alpha-glucosidase, an enzyme involved in starch digestion; SCXB the basic helix-loop-helix transcription factor; COL9A2 is a type IX collagen mostly known from hyaline cartilage; and CSPG5 is the chondroitin sulfate proteoglycan 5 (neuroglycan C). **(e)** Cervical stroma in mid-pregnancy labeled with BrdU. Note small dense nuclei with very few BrdU labeled cells. **(f)** Cervical stroma in late pregnancy labeled with BrdU. The cell nuclei are less dense and there are abundant cells labeled with BrdU, consistent with the finding that proliferation related genes are preferentially expressed in late pregnancy

In the comparison from non-pregnant and mid-pregnant cervices, 195 genes cross the operational criterion from being repressed to being expressed. Figure 2a shows the expression levels of genes operationally non-expressed in estrus but expressed in mid-pregnancy. Two genes stand out, CLCA1 and PLA2G10 (see Supplementary Table S2). CLCA1 is a component of Ca^2+^ sensitive chlorine channels and is involved in mucus production in other organs [42]. PLA2G10 is a phospholipase catalyzing the rate-limiting step in prostaglandin synthesis [43]. The other highly expressed and induced genes were: two members of the sodium-dependent decarboxylase transporters (SLC13A2 and SLC36A2), IGFBP1, as well as a tumor necrosis factor, TNFSF11.

There are 428 genes expressed during estrus but operationally OFF at mid-pregnancy. Figure 2b shows their expression level during estrus. There are 11 genes highly expressed during estrus but operationally turned OFF in mid-pregnancy with a discontinuity of lower expression level (big bold arrow in Fig. 2b). The most highly expressed gene turned off in pregnancy is interleukin 1a (IL1A). The other genes are TMPRSS11D, a trans-membrane serine protease involved in gland secretory activity, interferon kappa, IFNK and an antagonist of IL1A, IL1RN and others more (see list in Supplementary Table S3). The gene ontology categories most over-represented are related to inhibition of cell proliferation.

### Gene expression changes in the cervix from mid- to late-pregnancy

In the transition to late term 195 genes are turned on (Fig. 2c). The strongest expressed gene, which was not expressed in mid-pregnancy, is called VISG8, V-set and immunoglobulin domain containing 8. Very little is known about its function (Supplementary Table S4). The second most expressed gene turned on toward term is SPC24, a kinetochore complex component, foreshadowing the result of the gene ontology analysis. The overwhelming majority of genes are related to enhancement of cell proliferation, suggesting a very active proliferative state of the guinea pig cervix at term. These conclusions are supported by the induction or strong up-regulation of genes known to promote or are associated with tumor growth. For instance, among the induced genes is FOXM1 and among the strongest up-regulated genes are ARG2, arginase type II and FGFBP1. The high expression of ARG2 [44–46], FGFBP1 [47, 48] and FOXM1 [49–53], are associated with several cancers.

To determine whether the late term cervical stroma has proliferating cells we injected guinea pigs in mid- and late-term with BrdU and harvested the tissue 24 h later (Fig. 2e and f). While at mid-term the nuclei of stromal cells are small and dense, in late term the cell nuclei are larger and less dense. BrdU stained cells are abundant in latter (Fig. 2f) consistent with the transcriptomic data suggesting higher proliferative activity toward late term.

In the transition to term 449 genes meeting the operational criterion of expression in mid-pregnancy are turned OFF toward term. Four of them have higher expression than the rest of the distribution (Fig. 2d). These are: MGAM, SCXB, COL9A2 and CSPG5 (Supplementary Table S5). MGAM is the maltase-glucoamylase or alpha-glucosidase, an enzyme involved in starch digestion; SCXB the basic helix-loop-helix transcription factor; COL9A2 is a type IX collagen mostly known from hyaline cartilage and CSPG5 is the chondroitin sulfate proteoglycan 5 (neuroglycan C). Repression of these genes suggests a change in the extra-cellular matrix composition during transition to term.

### Expression dynamics of candidate genes

#### Steroid receptors

**Figure 3.**
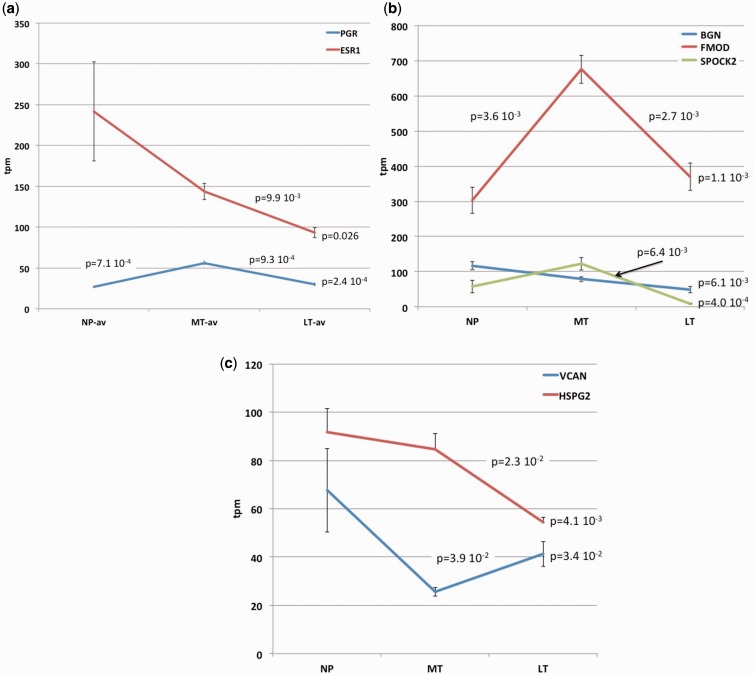
Expression dynamics of steroid receptors and proteoglycans. **(a)** Expression of ESR1 and PR. ESR1 is high during estrus and is steadily declining toward parturition. PGR is up-regulated in mid-pregnancy but returns to pre-pregnancy levels toward term. **(b)** mRNA expression dynamics of small proteoglycan genes: FMOD, testican 2 (SPOCK2) and BGN. **(c)** mRNA dynamics of large proteoglycan genes: VCAN and perlecan (HSPG2). Values at the right of each graph give the *P*-value of an ANOVA test including all three samples; the valued above the graphs give *t*-test based *P*-values for two-tailed pairwise comparisons

During estrus the expression of estrogen receptor, ESR1, is high (>240 TPM) and is decreasing to <150 TPM in mid-pregnancy and further decreases to <100 TPM toward term. In contrast, the progesterone receptor (PR) mRNA, PGR, is low during estrus (27 TPM) and rises to a value of 56 TPM in mid-pregnancy but falls close to pre-pregnancy levels of 30 TPM toward the end of pregnancy (Fig. 3a).

#### Extracellular matrix

The dominant collagen mRNA in the guinea pig cervix is type 1 collagen, COL1A1 and COL1A2. Expression is high for both type I collagen genes, ∼5000 TPM and 2000 TPM, respectively, and does not change among stages of the reproductive cycle. COL3A1, coding for collagen III identified in human cervix, was not mapped in our transcriptome data.

The dominant small proteoglycan is fibromodulin (FMOD) (Fig. 3b), which is known to associate with type I collagen and is also able to sequester TGF-beta in the extracellular matrix. FMOD shows a 2.2-fold increase from estrus to mid-pregnant stage and then a 0.55-fold decrease toward term, returning to about the same level as in estrus. A similar dynamics is observed for another small proteoglycan, testican 2, SPOCK2, although at lower levels of expression. It increases 2.1× from estrus to mid-pregnancy and toward term is almost completely suppressed, going from 122 TPM at MT to 9 TPM at LT (0.07-fold change). Repression of both FMOD and SPOCK2 transcription may play a role in extracellular matrix remodeling in preparation for parturition. In addition, the expression dynamics of FMOD suggests lower efficiency of TGF-beta signaling during pregnancy than before and toward term.

We found substantial expression for two large proteoglycans, versican (VCAN) and perlecan (HSPG2, heparan sulfate proteoglycan 2) (Fig. 3c). VCAN decreases from estrus to mid-pregnant by 0.38-fold and then increases 1.6-fold again toward term. In contrast, HSPG2 is high in estrus but declines slowly toward term to 59% from its value before pregnancy.

**Figure 4.**
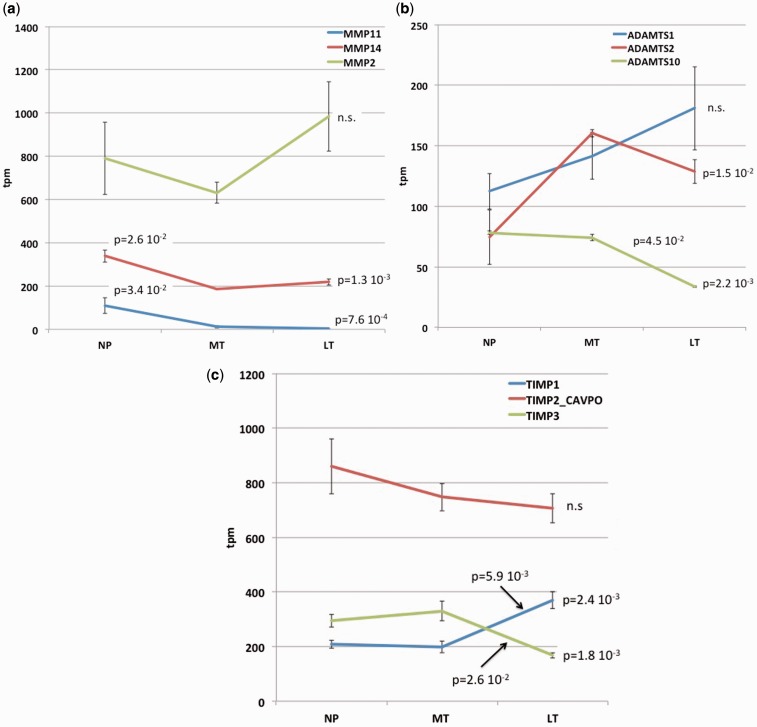
Expression dynamics of proteases. **(a)** mRNA expression of matrix metalloprotease genes. **(b)** mRNA expression of genes coding for ADAM metallopeptidases with thrombospondin motif, ADAMTS metallopeptidases. **(c)** mRNA expression of genes coding for inhibitors of matrix metallopeptidases, TIMPs. Values at the right of each graph give the *P*-value of an ANOVA test including all three samples; the valued above the graphs give *t*-test based *P*-values for two-tailed pairwise comparisons

The dominant matrix metallopeptidase mRNA is MMP2 (Fig. 4a), which is known to break down collagen IV and gelatin. There is an apparent increase in expression of MMP2 between mid- and late-term, but the statistical support is weak (*P* = 0.056). The next highest expression was found for MMP14, which is activating MMP2 and is likely membrane bound. MMP14 expression in estrus is ∼340 TPM and decreases to mid-pregnancy by 0.55-fold (*P* = 0.016) and shows a slight increase of 1.18-fold toward late term (*P* = 0.05). In contrast, MMP11 has a moderate expression in estrus (109 TPM), decreases toward mid-pregnancy by 90% (0.1-fold) and is barely above the operational threshold for expressed genes at term (3.75 TPM). This MMP11 is not effective in extracellular matrix breakdown, but is reported to cleave protease inhibitor A1AT, which is not included in our mapped transcripts.

Among the ADAM metallopeptidases with thrombospondin motif, ADAMTS metallopeptidases, ADAMTS1 and -2 are most expressed. ADAMTS1 shows a slow but insignificant increase from non-pregnant to term, while ADAMTS2 is increasing from non- to mid-pregnant and decreases at late term (Fig. 4b).

The most expressed inhibitor of matrix metallopeptidase is TIMP2, which is highly expressed during estrus and shows a slow but insignificant decline toward term (Fig. 4c). The only highly expressed protease inhibitor that shows a significant increase from mid- to late-term is TIMP1 (1.9×, *P* = 0.006). TIMP1 is associated with cell proliferation and may have anti-apoptotic effects consistent with other results suggesting proliferation in later term cervix (see above and Fig. 2e and f). In contrast, TIMP3 shows a 50% reduction in expression between mid- and late-term (0.51×, *P* = 0.026).

#### Signaling

**Figure 5.**
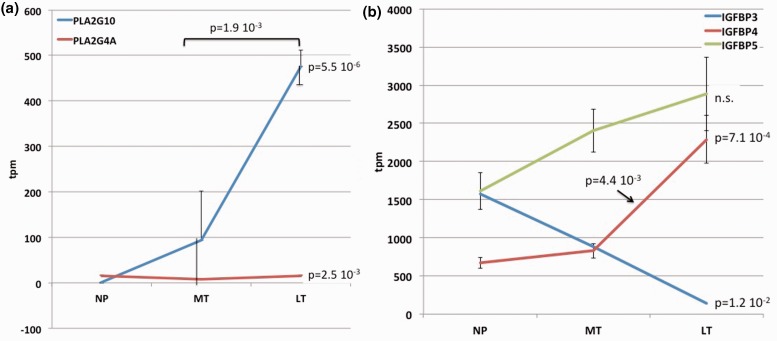
Expression dynamics of genes related to prostaglandin and IGF signaling. **(a)** mRNA expression of genes coding for PLA2 gamma 4A and 10, PLA2G4A and PLAG210. Note the steep increase in the expression level of PLA2G10, which suggests an increased paracrine expression of prostaglandin, since PLA2 is catalyzes a rate-limiting step in prostaglandin biosynthesis. **(b)** mRNA expression of genes coding for insulin like growth factor binding proteins, IGFBP3, IGFBP4 and IGFBP5. Values at the right of each graph give the *P*-value of an ANOVA test including all three samples; the valued above the graphs give *t*-test based *P*-values for two-tailed pairwise comparisons

Prostaglandin signaling, in particular PGE_2_ and PGF_2a_, is associated with many aspects of female reproductive physiology including parturition [54]. A rate-limiting step in prostaglandin synthesis is the liberation of arachidonic acid from phospholipids by phospholipase A and C. In the guinea pig cervix we find a substantial increase in PLA2G10 just before parturition (Fig. 5a). Interestingly, the expression of cyclo-oxygenase II, catalyzing another key step in prostaglandin synthesis, is not increased toward term.

The expression of IGF signaling molecules came to our attention though the conserved up-regulation of IGFBP4 in both human and guinea pig cervices (see below). mRNA expression of IGF1 and IGF2 toward term follow different trends, with IGF1 being up-regulated (1.57×, *P* = 0.0074) and IGF2 down-regulated (0.66×, *P* = 0.0038). In contrast, there is no substantial change in IGF receptor mRNA expression.

There is high expression of most IGF binding proteins, with three having a dramatic regulatory change (Fig. 5b). IGFBP5 is the most expressed IGFBP overall but does not show a significant difference between pregnancy stages. IGFBP4 shows the most dramatic up-regulation (2.75×, *P* = 0.0022). In contrast, IGFBP3 is expressed at about the same level as IGFBP5 at estrus and then shows a gradual decline during pregnancy with very low expression at term (from 1500 TPM during estrus to 140 TPM at term). These results could point to an attenuation of IGF2 signaling in the term cervix of the guinea pig because two binding proteins are increasing, and IGF2 expression is decreasing (but IGF1 mRNA is increasing).

## DISCUSSION

In this study we addressed two questions. First, we investigated whether FPW in primates and guinea pigs has evolved independently or whether these are homologous features. Based on a parsimony reconstruction of ancestral character states we inferred that FPW in guinea pigs evolved independently of that in primates (see Fig. 1). Second, we investigated the gene regulatory dynamics during pregnancy in the uterine cervix using RNAseq to assess how CRM is regulated in the presence of sustained progesterone levels in the guinea pig. Our data suggest two mechanisms involved in CRM, down-regulation of PR expression and, potentially, the intracervical activation of prostaglandin synthesis, that are discussed in more detail below.

### Evolution of functional progesterone withdrawal

Here, we use the term FPW as meaning a situation in which the circulating progesterone levels do not substantially fall prior to parturition. At this point, we do not ascribe a specific mechanistic meaning to the term FPW. The reason is that it is still unclear how different the regulation of CRM is between species with systemic progesterone withdrawal and those without systemic progesterone withdrawal; with some authors clearly favoring the view that the mechanisms of CRM are conserved between mouse and human [55], while the question still remains whether sustained high levels of circulating progesterone have consequences for the regulation of CRM and parturition in general [56].

The question whether FPW in guinea pigs and primates is homologous, requires a detailed investigation because the guinea pig represents a basal lineage among the rodents and FPW is widespread in Euarchonta, the clade consisting of primates, tree shrews and flying lemurs (for references see above). It is thus possible that FPW could have been an ancestral state within the Archontoglires, the clade uniting primates and rodents and their close relatives. In Euarchonta, the most basal lineage with data on progesterone levels at parturition is the northern tree shrew (*Tupaia belangeri*). For this species, FPW has been documented, and thus it is possible that FPW can be quite old.

Data about progesterone levels at parturition in non-model organisms are sparse, but sufficient data of the basal lineages of the Euarchontoglires are available to allow a unique parsimony reconstruction (Fig. 1). The conclusion that FPW evolved twice independently is based on the serum progesterone withdrawal in rabbits and Sciuridae (here represented by ground squirrel and woodchucks), which are two lineages more basal than the most recent common ancestor of guinea pigs and other rodents (Fig. 1). The outgroup of the Euarchontoglires is Lauresiatheria, which is a large group containing as diverse animals as bats, horses, cows, hedgehogs and carnivores and others. We did not perform a detailed analysis of data on circulating progesterone levels during pregnancy in this group, but we are not aware of any member of this group to show FPW. It is widely acknowledged that sheep, cow, pig, horse, dog and cat have circulating serum progesterone withdrawal at parturition. We thus infer that the ancestral state for Euarchontoglires is withdrawal of serum progesterone. Based on these data we consider the conclusion that primates and guinea pigs evolved FPW independently as robust.

### Physiology of cervix remodeling in guinea pig

An intriguing finding about guinea pig CRM was reported by Rodríguez *et al.* [57]. The authors quantified the density of PR and ESR1 positive cells in the cervix and the uterus and reported a 50% drop in the number of PR positive cells in the sub-epithelial portion of the cervix, i.e. the cervical stroma, 1–2 days before parturition. The authors also found a negative correlation between PR and collagen integrity, suggestive of a causal link between loss of PR expression and CRM. This finding is intriguing as it provides a mechanistic model for FPW, at least for the cervical stroma (no comparable drop in PR expression was found in the myometrium). Our data are consistent with that of Rodríguez *et al.* as we find also a 50% drop in PR mRNA expression between mid- and late-pregnancy. Interestingly, though, no comparable decrease in PR mRNA abundance was reported for human cervix [17, 58, 59]. Rodríguez *et al.* also reported only small if any increase in ESR1 expression toward term. In our data we find a modest 0.7×, but significant (*P* < 0.01) decrease in ESR1 mRNA abundance, which is similar to the 0.84× decrease of ESR1 mRNA in human cervix [17].

Another factor implicated in guinea pig CRM is phospholipase A2 (PLA2). PLA2 catalyzes a rate-limiting step in prostaglandin synthesis by liberating arachidonic acid from phospholipids [43]. Prostaglandin E_2_ and F_2-alpha_ have been shown to stimulate collagenase activity in confluent guinea pig cervical cell culture [60], and it is also long established that prostaglandins can induce cervical ripening [1]. PLA2 activity increases ∼20-fold between non-pregnant to later term cervix extractions [61]. The PLA2 activity measured by Rajabi and collaborators was mostly due to the 85 kD cytosolic PLA2 (cPLA2), but contributions of lower molecular weight secreted PLA2 was also detected, with a distinct protein peak around 20 kD [61]. Surprisingly, no evidence for different protein levels was found between non-pregnant and term cervices by Rajabi and Cybulsky but this was based on protein gel band intensities. In our data, the mRNA abundance for cPLA2 (PLA2G4A) was at moderate but significant 15 TPM at late term, and at the same level for cervices of non-pregnant guinea pigs. In mid-term, the level was lower at ∼7 TPM, which is 2× different from late-term (*P* = 8.88 × 10^−^^4^). However, the most dramatic change we found was in a low-molecular weight secreted PLA2 (PLA2G10, 18 kD), which is not expressed before pregnancy and increases to 200 and 400 TPM at mid- and late-term pregnancy, respectively. Hence, our data are consistent with the results of Rajabi *et al.* with respect to protein levels of cPLA2 in non-pregnant and late term cervices, but also shows a dramatic induction of a secreted PLA2, PLA2G10, which is not expressed before pregnancy. These data suggest an active role of prostaglandin signaling in normal CRM in guinea pigs.

In guinea pigs, CRM is in part achieved through collagen degradation effected by collagenases [62, 63]. Rajabi *et al.* reported a 5-fold increase in procollagenase activity from Day 50 to parturition. In our data the highest RNA expression level was found for MMP2, with 1000 TPM in late pregnancy and a 1.6-fold increase from mid-term to late-term. Among the ADAMTS metalloproteases, the highest expression was found to be ADAMTS1, with a 1.28× increase relative to mid-term and a final 181 TPM. The highest fold increase was recorded for ADAMTS8 with a 6.7× increase consistent with the finding of Hassan *et al.*, who reported a 2.9× increase in human cervix [17]. The final RNA abundance of ADAMTS8 (14 TPM), however, was more than one order of magnitude lower than that of ADAMTS1 (181 TPM) and even smaller than the 1000 TPM found for MMT2. This comparison illustrates the danger of sole reliance on fold change measures to identify biologically important factors.

Overall expression of type I collagen, collagenases and inhibitors of matrix metallopeptidases do not show a clear picture that would explain extracellular matrix remodeling. In contrast, the proteoglycan expression suggests a role in CRM with increased expression of the large proteoglycan VCAN. Small proteoglycans, FMOD, testican, SPOCK2 and biglycan (BGN), in contrast, all show considerable decrease toward term. FMOD is interacting with type I and type II collagens. BGN plays a role in assembly of collagen fibrils, and testican is also involved in extracellular matrix organization.

### Comparison to human cervix

There are no data available to directly compare the results reported in the guinea pig with those in humans. The only data set we are aware of from humans that has some relationship to the guinea pig data presented here is Affymetrix based data from ripe and unripe cervices at term [17]. Ideally, both human and the guinea pig tissues should be obtained from corresponding stages of gestation. However, there are methodological and ethical considerations to prevent such a comparison. Obtaining cervix samples from women with ongoing gestations in the mid-trimester is not feasible because of the risk of causing a spontaneous abortion. On the other hand, the diagnosis of cervical ripening in the guinea pig is difficult. Changes in collagen have been quantitated using a non-invasive approach, which is not widely available to investigators [64, 65]. Therefore, a large number of guinea pigs at term would need to be euthanized to obtain material that would include both ripe and unripe cervices.

One way of relating the observations made in this study to data derived from humans is to assume that an unripe cervix at term is a cervix that did not make the transition from mid-pregnant state to a compliant, ripe cervix. From this perspective, an unripe cervix at term is probably closer to a mid-gestation pregnant cervix than to a ripe cervix. This assumption can be challenged; yet, despite the limitations, we believe that this comparison may offer insight into the comparative biology of cervical ripening, which may be difficult to gather otherwise at this time.

Another methodological issue is the comparison of transcriptomic data derived from microarrays (Affymetrix) and RNAseq data. Studies in humans have been based on Affymetrix technology [17], while our data were obtained using RNAseq. Whether and how Affymetrix and RNAseq data can be compared relies upon whether the different scales on which Affymetrix and RNAseq measures reside affect our conclusion. A key question when exploring this comparison is whether, on average, the direction of changes in gene expression between the two data sets corresponded. To address this, we first only considered statistically significant changes from the human data, and asked whether the RNAseq data (for the same genes) varied in the same direction. We considered fold changes estimated by Affymetrix and RNAseq technology. Fold changes are scale less quantities and thus, the original scales of RNA abundance measures became irrelevant, assuming their approximate linearity. The comparisons between the human and guinea pig samples were determined by calculating the correlation among the fold change data. This essentially tested for congruence of the direction of change rather than the magnitude of fold change. The only scenario where the technological difference would matter is if a change in one direction detected by Affimetrix technology would consistently lead to a signal in the opposite direction in RNAseq data. A substantial degree of methodological pessimism would be required to accept this scenario.

We find that there is the potential of shared conserved regulatory pathways—most notably, the down-regulation of ESR1 and Polybromo 1 (PBRM1) as well as the up-regulation of IGFBP4, and the increased expression of VCAN. The other changes reported in the comparison of human ripe and unripe cervical tissue are not found in our comparison of mid- and late-gestation samples from guinea pig. There are methodological limitations that could explain these differences, and thus, the interpretation needs to proceed cautiously. At the very least, these data do not support a strong similarity of gene regulatory dynamics in humans and guinea pigs (Supplementary Fig. S1). Hassan *et al.* reported 89 genes with significant differences between ripe and unripe cervices. Most of these are up-regulated (*n* = 81). Of these 89 genes 76 where detected or expressed in our guinea pig samples. The correlation between the log 2-fold differences in the Hassan data and the log 2-fold expression data from guinea pig is low, *r* = 0.054. We then determined the set of genes that have a fold change in the same direction as in human samples and are significantly different between MT and LT (concordant genes). The list of concordant genes is short, comprising only nine genes: ESR1, PBRM1, SIGLEC1, CALD1, TPM1, IGFBP4, PLN, COL4A2 and VCAN. In contrast, there are 27 genes that are discordant, found to differ in the opposite direction between unripe and ripe human cervices at term and between MT and LT guinea pig cervix.

More comparative data are necessary to assess the existence of conserved core-regulatory mechanisms shared among placental mammals, or at least among the Archontoglires, the clade combining humans and mice. Any mechanistic interpretation of transcriptomic data, however, will require follow-up studies to exclude the possibility that the changes in RNA abundance are due to changes in tissue composition, e.g. through the recruitment of leucocytes.

## CONCLUSIONS AND IMPLICATIONS

1. Lack of systemic progesterone withdrawal at parturition evolved independently in the primate and the guinea pig lineages.
2. In guinea pig CRM at term is associated with changes in proteoglycan expression and less so with changes in the expression of collagen or collagenases.
3. We confirm previous evidence for a down-regulation of PR mRNA expression in the guinea pig cervix at term, a feature that has not been described in humans.
4. Potentially conserved mechanisms of CRM include down-regulation of ESR1 and the nuclear receptor PBRM1 as well as the up-regulation of VCAN and IGFBP4.

## SUPPLEMENTARY DATA

Supplementary data are available at *EMPH* online.

## FUNDING

This research was supported, in part, by the Perinatology Research Branch, Division of Intramural Research, *Eunice Kennedy Shriver* National Institute of Child Health and Human Development, National Institutes of Health, Department of Health and Human Services (NICHD/NIH); and, in part, with Federal funds from NICHD, NIH under Contract No. HSN275201300006C.

**Conflict of interest**: None declared.

## Supplementary Material

Supplementary Data
