# Supplementary material for: Evidence for independent evolution of functional progesterone withdrawal in primates and guinea pigs
Source: Evol Med Public Health. 2013 Dec 3;2013(1):273–88. doi: 10.1093/emph/eot022 (PMC3875370; doi:10.1093/emph/eot022)
Supplement: Supplementary Data [file supp_2013_1_273__index.html]

Supplementary Data 

# Evidence for independent evolution of functional progesterone withdrawal in primates and guinea pigs

## Supplementary Data

files

**Files in this Data Supplement:**

- Supplementary Data - zip file
