# Supplementary material for: Evidence for independent evolution of functional progesterone withdrawal in primates and guinea pigs
Source: Evol Med Public Health. 2013 Dec 3;2013(1):273–88. doi: 10.1093/emph/eot022 (PMC3875370; doi:10.1093/emph/eot022)
Supplement: Supplementary Data [file supp_eot022_eot022_supp.zip › Nnamani_et_al-Supplementary_Material.pdf]

Supplementary Material

Evidence for the independent evolution of "functional progesterone withdrawal" in primates and guinea pigs:
a comparative transcriptomic study.

Mauris C. Nnamani, Roberto Romero, and Günter P. Wagner
Yale Systems Biology Institute
and
Department of Ecology and Evolutionary Biology
Yale University
Perinatology Research Branch, NICDH, NIH
Department of Obstetrics and Gynecology
Wayne State University

Suppl. Table 1: fetal measurements to assess the status of pregnancy at time of tissue harvesting.

Animal ID	Weight of Animal (g)	weight of embryos (g)	Average weight of Embryos (g)	Head size SPL (mm)	Average head size SPL (mm)	[1,2]Estimated mean fetal weight (g)	[3,4]Estimated gestation period (days)	Average estimated gestation period (days) ± Stdev	Number of pups
GpLT 001	1140	106.7, 88.6, 85.3	93.53	44.4, 43.3, 46.7	44.8	98.7	66.05	65.2 ± 1.2	3
GpLT 002	1120	103.7, 87.4	95.55	44.3, 45.2	44.75	98.5	63.9		2
GpLT 003	1282	67, 66.1, 82.1, 68.3, 75.1	71.72	40.8, 40.4, 42, 43.4, 41.3	41.58	84.6	65.7		5
GpMT 586	1135	25	25	26.8	26.8	17.3	43	43.6 ± 0.5	1
GpMT 587	1201	19.1, 18.9, 18.9, 14.1, 20.7	18.34	26.9, 23.8, 26.7, 26.2, 26.6	26.04	16.7	43.8		5
GpMT 588	1364	26.7, 24.8, 21.9, 27.1	25.13	28.9, 28.5, 26.2, 23.3	26.73	19.5	43.9		4 (one fetus appeared to have deteriorated within the uterus)
GpNP 538	499	N/A							
GpNP 405	615	N/A							
GpNP 406	573	N/A							

[1,2] Thomas and Lowy, 1982; Ibsen, 1928; [3,4] Draper, 1920; Kihlstrom, 1985

Suppl. Table 2: genes not expressed in cervix during estrus but expressed in mid pregnancy with average expression level > 3 tpm in mid pregnancy and a p-value of <0.05 by one sample t-test of the log transformed values at mid pregnancy.

Ensembl Gene ID	Associated Gene Name	MT-AV [tpm]	T-VALUE MT	P-VALUE MT
ENSCPOG00000014947	CLCA1	204.0636269	10.77600695	0.004250971
ENSCPOG00000027338	PLA2G10	201.039972	7.796141971	0.008028803
ENSCPOG00000009792	SLC13A2	87.76467567	57.0962228	0.000153305
ENSCPOG00000020697	SPDEF	76.83951878	29.07151728	0.000590561
ENSCPOG00000026955	KLK1_CAVPO	69.20396696	6.371800642	0.011878225
ENSCPOG00000009753	LRRC26	60.26485144	4.627538709	0.021831244
ENSCPOG00000000599	VSIG2	59.81765517	26.31689831	0.000720379
ENSCPOG00000021732	FGFBP1	59.67672089	4.609091438	0.021994961
ENSCPOG00000015712	KIAA1324	51.23971544	9.452106242	0.005504211
ENSCPOG00000000344	SULT1C2	46.2403785	22.72569242	0.000965332
ENSCPOG00000005916	ERN2	41.72706999	6.756769424	0.010604775
ENSCPOG00000012000	PACSIN1	29.19563225	17.28196027	0.001665749
ENSCPOG00000008306	SLC36A2	29.10696902	32.95321703	0.000459806
ENSCPOG00000004659	TNFSF11	29.01362336	17.16895153	0.001687638
ENSCPOG00000011137	WDR72	24.80829072	17.98171251	0.001539214
ENSCPOG00000009423	BHLHA15	20.14012663	14.41917506	0.002387644
ENSCPOG00000011786	NXPE2	17.95903593	33.45779482	0.000446061
ENSCPOG00000001636	FBP1	17.94589195	15.22323822	0.00214366
ENSCPOG00000010407	FXYD4	15.96129655	5.452094812	0.016016812
ENSCPOG00000006412	NRCAM	15.63200424	32.59084171	0.000470074
ENSCPOG00000014607	MYB	15.29113397	28.1378433	0.000630328
ENSCPOG00000009706	SLCO1A2	15.25001697	9.653807266	0.005280202
ENSCPOG00000022244	KNDC1	14.51271063	34.67864408	0.000415245
ENSCPOG00000001693	GPC6	14.253491	14.61325186	0.002325084

ENSCPOG00000006519	LECT1	12.96579024	6.945537816	0.010053198
ENSCPOG00000026904	MYO7A	12.23687874	7.307501277	0.009108301
ENSCPOG00000004457	CCDC19	12.15414086	4.985049381	0.018981785
ENSCPOG00000015654	LSAMP	12.13555213	9.206989584	0.005796044
ENSCPOG00000011138	UNC5CL	11.71655031	11.88100281	0.003504923
ENSCPOG00000014301	KCNK12	11.67406153	14.60340627	0.002328199
ENSCPOG00000015264	FA2H	11.66739132	21.14035384	0.001115041
ENSCPOG00000004500	TMEM221	10.97509442	5.226233571	0.017358287
ENSCPOG00000024031	GRM8	10.65671423	12.34008713	0.00325148
ENSCPOG00000020319	KLHDC8A	10.30968789	8.882799669	0.006218828
ENSCPOG00000025862	AZGP1	10.08416806	3.723628998	0.032576311
ENSCPOG00000021963	CHST4	9.973413429	11.53343966	0.003716965
ENSCPOG00000012663	STYK1	8.874898368	7.651774343	0.008327025
ENSCPOG00000000793	CHST8	8.75536275	6.244145017	0.012350835
ENSCPOG00000021148	TSPO2	8.249937465	6.259229475	0.012293551
ENSCPOG00000010139	OLAH	8.077416267	3.094789682	0.04523235
ENSCPOG00000008402	ABCA3	8.055744929	14.39136486	0.002396815
ENSCPOG00000001747	CDH7	8.00498954	9.507155011	0.005441688
ENSCPOG00000009920	BDNF_CAVPO	7.811124349	33.26534788	0.00045123
ENSCPOG00000002132	O3FAR1	7.681815258	7.745837593	0.008130888
ENSCPOG00000021703	RBFOX3	7.433713273	4.20161411	0.026123098
ENSCPOG00000023925	UNC5A	7.188153655	4.128946816	0.026976953
ENSCPOG00000021930	NOTO	7.091161662	14.425751	0.002385484
ENSCPOG00000008243	TMEM74	7.026684737	4.719442433	0.021041655
ENSCPOG00000002686	SLC26A7	6.974492714	4.632797321	0.021784901
ENSCPOG00000002984	PPP2R2C	6.948554389	3.923467166	0.029623783
ENSCPOG00000005761	FAM83E	6.81586658	13.17440015	0.002856108
ENSCPOG00000005182	CARD14	6.750460231	5.235206623	0.017301885

ENSCPOG00000021576	MFSD6L	6.684817361	9.793723977	0.005132709
ENSCPOG00000014697	TMEM139	6.67958663	4.591743278	0.022150572
ENSCPOG00000023622	FAM43B	6.646880224	8.820748591	0.006304977
ENSCPOG00000020016	OXTR	6.632991591	11.06340659	0.004035612
ENSCPOG00000001651	MYOT	6.605670951	5.53342909	0.015571023
ENSCPOG00000023177	SLC16A6	6.484216529	5.822629076	0.014125952
ENSCPOG00000026830	ATP2C2	6.257959812	3.311297847	0.040180775
ENSCPOG00000024661	FOXA3	6.241830281	2.958378237	0.048893547
ENSCPOG00000021412	GABRE	6.196608953	15.92023372	0.001961147
ENSCPOG00000014997	LRRC4C	5.814795876	8.013642256	0.007608656
ENSCPOG00000005296	F7	5.548177046	5.695320318	0.014736571
ENSCPOG00000012705	SLC4A5	5.528130194	3.485764588	0.036679704
ENSCPOG00000007999	FAM65B	5.519077144	3.819924401	0.031102706
ENSCPOG00000014526	GPR20	5.362742254	3.045137597	0.046518253
ENSCPOG00000006861	PTGDR	5.356570294	2.923231169	0.049905094
ENSCPOG00000022451	BIK	5.342310437	5.524301674	0.015620131
ENSCPOG00000022756	CA13	5.020668893	3.010478482	0.047446993
ENSCPOG00000009012	GRAMD2	5.002697196	10.0339913	0.004893396
ENSCPOG00000006755	MCOLN2	4.817758264	10.66931835	0.004335304
ENSCPOG00000013818	LIPH	4.452434225	2.976974406	0.04837011
ENSCPOG00000008861	DGKI	4.24177782	3.152311703	0.043805227
ENSCPOG00000003316	NRXN1	4.149429889	3.006321741	0.047560151
ENSCPOG00000020150	TMEM178B	4.123368664	3.252144067	0.041477237
ENSCPOG00000009122	Bfsp2	4.062616414	4.469528582	0.023293993
ENSCPOG00000021122	ZBTB42	4.01794575	4.772383305	0.020605707
ENSCPOG00000026453	KBTBD11	3.991760214	4.412770163	0.023854596
ENSCPOG00000011482	MTMR7	3.9585898	3.83676985	0.030854895
ENSCPOG00000008280	ESRRG	3.929286917	11.98175694	0.003446831

ENSCPOG00000002997	CYP4X1	3.900628331	9.863040991	0.005061905
ENSCPOG00000001518	PPARGC1A	3.882643392	4.641614914	0.021707518
ENSCPOG00000012755	SORCS1	3.855453085	3.895372828	0.030014783
ENSCPOG00000010494	KLRG1	3.808041199	7.350784624	0.009004224
ENSCPOG00000010650	FAM65C	3.611531764	3.228347703	0.042015749
ENSCPOG00000003556	TDRKH	3.481322606	4.038340859	0.028099769
ENSCPOG00000001158	PAG1	3.291018235	2.957183915	0.04892744
ENSCPOG00000005997	TSKS	3.264325833	6.329142766	0.012033127

Supplementary Table 3: genes not expressed in mid pregnancy cervix but expressed before pregnancy with average expression level > 3 tpm before pregnancy and a p-value of <0.05 by one sample one tailed t-test of the log transformed values in non-pregnant cervix.

Ensembl Gene ID	Associated Gene Name	NP-AV [tpm]	T VALUE NP	P VALUE NP
ENSCPOG00000026158	IL1A_CAVPO	172.1337951	4.234782924	0.025746424
ENSCPOG00000010047	TMPRSS11D	130.3707772	4.921357299	0.019447806
ENSCPOG00000000866	IFNK	104.5894392	3.335603227	0.039664919
ENSCPOG00000023835	CRCT1	97.37797428	3.248692343	0.041554733
ENSCPOG00000019858	NOXO1	94.81162273	5.633087501	0.015049346
ENSCPOG00000009033	RHCG	78.72404354	3.906224768	0.029862868
ENSCPOG00000008107	RSAD2	66.48682566	3.256699071	0.041375287
ENSCPOG00000008700	IL1RN	61.84558509	4.695066196	0.021246947
ENSCPOG00000014264	SPINK5	59.12181129	4.951693311	0.01922377
ENSCPOG00000005946	DHRS9	54.57732201	3.599425997	0.034631001
ENSCPOG00000001205	IVL	53.26558754	3.469174475	0.036993291
ENSCPOG00000002046	CXCL10	19.71037868	3.677030695	0.033325963
ENSCPOG00000003871	VASH2	15.07713462	6.858454993	0.010302226
ENSCPOG00000005926	SPC25	14.97406531	8.668835603	0.006523546
ENSCPOG00000001375	FAM159B	14.28228604	5.885476645	0.013838218
ENSCPOG00000014147	MKI67	13.37048568	5.676152351	0.014831876
ENSCPOG00000005291	RASL10A	12.68145582	5.071711224	0.018373642
ENSCPOG00000004546	CCL22	12.21328909	2.927471154	0.049781496
ENSCPOG00000003286	AURKB	10.76144597	7.011992797	0.009869124
ENSCPOG00000008028	NEFH	10.6330052	11.77747995	0.00356615
ENSCPOG00000003953	RSPO1	10.4768371	5.132937636	0.017961122
ENSCPOG00000005646	IL18BP	10.21198144	3.157065204	0.043690186
ENSCPOG00000009201	PLEKHS1	10.13623021	4.921786618	0.019444609

ENSCPOG00000008312	KIF20A	10.03281902	6.928415132	0.010101452
ENSCPOG00000009002	BUB1B	9.797333026	7.266234718	0.009209202
ENSCPOG00000026951	FAM83D	9.778919972	9.435746886	0.005522998
ENSCPOG00000000236	SESN2	9.60494341	16.11976301	0.001913174
ENSCPOG00000020676	CDK5R1	9.524839161	8.348052423	0.007023809
ENSCPOG00000022312	SPC24	9.520936563	7.55730245	0.008531183
ENSCPOG00000015373	FOXM1	9.376064988	5.891005634	0.013813321
ENSCPOG00000007305	CEP55	9.2665531	4.650403505	0.02163079
ENSCPOG00000026600	ANKRD37	9.250363922	11.61147482	0.003667719
ENSCPOG00000007875	SPINK6	9.216843167	3.315303543	0.040095097
ENSCPOG00000001228	CDCA8	9.068128213	5.888849166	0.013823024
ENSCPOG00000008838	RBM24	8.869063309	3.786654407	0.031600697
ENSCPOG00000008241	MEX3B	8.457117196	5.121619842	0.018036343
ENSCPOG00000007302	DUOX1	8.329923455	4.839092197	0.020075024
ENSCPOG00000004318	KIF23	8.020212797	4.631994426	0.021791968
ENSCPOG00000014374	CENPF	7.907604644	6.258028131	0.012298099
ENSCPOG00000019656	ARHGEF19	7.872766402	3.204483358	0.042565927
ENSCPOG00000001781	WIF1	7.860712734	3.172891879	0.043310265
ENSCPOG00000006055	GTSE1	7.556329143	13.29864035	0.002803438
ENSCPOG00000010155	NCAPH	7.529681579	4.757029585	0.020730763
ENSCPOG00000000523	RIMS1	7.263606812	12.28962985	0.003277972
ENSCPOG00000024081	OIP5	7.17629632	4.651687103	0.021619617
ENSCPOG00000004304	POLE2	7.080525276	9.503378071	0.005445943
ENSCPOG00000001241	ZWILCH	7.072694552	6.963554934	0.010002792
ENSCPOG00000009232	KCNS3	7.024071661	16.44166216	0.001839403
ENSCPOG00000016399	SNORD14	7.021589552	3.568602285	0.035169915
ENSCPOG00000025118	CENPA	6.922085908	7.217676001	0.009330075
ENSCPOG00000010632	KIFC1	6.909582536	5.268850234	0.017092797

ENSCPOG00000014591	MELK	6.784333271	7.70347807	0.008218354
ENSCPOG00000003867	ADAM12	6.779371637	3.718835784	0.032652289
ENSCPOG00000011049	PTGES	6.752202949	4.804051533	0.020351243
ENSCPOG00000004744	ECT2	6.663415785	3.976446582	0.028906339
ENSCPOG00000005838	NDC80	6.614779027	6.450647564	0.011599586
ENSCPOG00000000878	HMMR	6.613479246	3.925961957	0.029589421
ENSCPOG00000003651	SRPX2	6.496214337	3.554363113	0.035422969
ENSCPOG00000022439	VSTM4	6.493756486	3.713041276	0.032744481
ENSCPOG00000008318	SGOL1	6.466235551	4.681834264	0.021359617
ENSCPOG00000013158	BUB1	6.461962491	5.643162604	0.014998048
ENSCPOG00000004603	ASPM	6.297726967	3.38876096	0.038569407
ENSCPOG00000004807	DLGAP5	6.291074437	4.031165536	0.028191582
ENSCPOG00000004203	CDKN3	6.290668105	3.905574886	0.029871934
ENSCPOG00000008248	DHTKD1	6.255526389	9.520843896	0.005426305
ENSCPOG00000015019	NUF2	6.173516621	3.960643305	0.029117676
ENSCPOG00000002934	SPAG5	6.112828031	3.903253576	0.02990435
ENSCPOG00000011684	TNFRSF4	6.105784569	6.237692913	0.012375457
ENSCPOG00000026208	WDFY4	5.956261602	2.952440438	0.04906238
ENSCPOG00000010952	B4GALNT3	5.954180232	9.298939482	0.005683923
ENSCPOG00000010218	COX4I2	5.927706797	15.27979818	0.002127921
ENSCPOG00000004066	BCL6B	5.896052362	6.209795194	0.012482763
ENSCPOG00000006691	GPR85	5.862210174	6.462605944	0.011558174
ENSCPOG00000014099	CASC5	5.842425506	6.699296005	0.010781644
ENSCPOG00000003710	EZH2	5.816153079	3.278625503	0.040889531
ENSCPOG00000009022	DUSP15	5.742360606	6.974036025	0.009973641
ENSCPOG00000019548	TNFRSF25	5.689054209	3.643596677	0.033879326
ENSCPOG00000007818	BCL2A1	5.64168662	4.247262334	0.025606755
ENSCPOG00000013206	CENPM	5.641264697	3.664429671	0.033532974

ENSCPOG00000027389	MYO1G	5.61968278	2.958351963	0.048894293
ENSCPOG00000001220	NCAPG	5.592540171	4.189084093	0.026267488
ENSCPOG00000003302	KIF20B	5.49824503	5.25275987	0.017192328
ENSCPOG00000005619	SOAT2	5.481414357	3.278456806	0.040893237
ENSCPOG00000023385	SNORA72	5.4677696	8.067192811	0.007510235
ENSCPOG00000004994	HJURP	5.439411675	5.352652399	0.0165879
ENSCPOG00000027699	SNORD62	5.405579318	3.109070538	0.044871906
ENSCPOG00000015316	TMEM37	5.378919861	4.065248555	0.027759318
ENSCPOG00000013130	PRR11	5.362142938	2.953369518	0.049035908
ENSCPOG00000025763	PTGER1	5.34895623	5.222137949	0.017384122
ENSCPOG00000014105	RAD51	5.333073656	4.016693583	0.028378094
ENSCPOG00000005607	S100A3	5.317926719	63.60546918	0.000123544
ENSCPOG00000004459	INHA	5.292309944	5.909233738	0.013731706
ENSCPOG00000027174	KIF18A	5.240581098	5.075792897	0.018345709
ENSCPOG00000011288	SCIMP	5.237489957	3.818312921	0.031126563
ENSCPOG00000009378	GBP6	5.17669917	10.8851522	0.004167207
ENSCPOG00000012532	TBX1	5.166069448	12.21607506	0.003317172
ENSCPOG00000008286	DBF4	5.164295868	7.705689547	0.008213753
ENSCPOG00000024101	MIA	5.143694195	7.377469271	0.008940938
ENSCPOG00000002037	ARHGAP11A	5.115629423	3.631638011	0.034080484
ENSCPOG00000006801	ITGA2B	5.11330147	4.275978942	0.025289536
ENSCPOG00000008606	GPR183	5.043369177	5.483391027	0.015843076
ENSCPOG00000004089	WASF1	4.971510903	5.064412343	0.018423747
ENSCPOG00000013678	NFKBID	4.955339048	5.222118546	0.017384244
ENSCPOG00000026161	SLC25A27	4.88832698	11.06391352	0.004035247
ENSCPOG00000024975	QPRT	4.742268849	3.908049182	0.029837438
ENSCPOG00000002187	TMOD4	4.687096371	5.360848477	0.016539704
ENSCPOG00000010752	RNF183	4.646964935	5.164864689	0.017751401

ENSCPOG00000003365	TMEM178A	4.489550061	4.770588293	0.02062027
ENSCPOG00000005635	WNT16	4.469338231	3.467493553	0.037025279
ENSCPOG00000023842	YJEFN3	4.445421392	3.159825181	0.04362359
ENSCPOG00000004072	USP44	4.436861365	17.00225565	0.001720721
ENSCPOG00000004015	CENPN	4.428592093	4.019198531	0.028345683
ENSCPOG00000017956	SNORND104	4.393417385	4.335596804	0.024649042
ENSCPOG00000015342	ARHGAP22	4.355478611	5.717619768	0.014626831
ENSCPOG00000009200	CLCN2_CAVPO	4.31885968	7.37474423	0.008947371
ENSCPOG00000023486	CCDC142	4.296556079	4.492723888	0.023070435
ENSCPOG00000004737	RCOR2	4.288877405	5.041421391	0.018582896
ENSCPOG00000018335	SNORA68	4.264596659	3.012472582	0.047392845
ENSCPOG00000025080	CHD5	4.208573289	3.573532906	0.035082898
ENSCPOG00000008754	SLC1A4	4.197315882	3.192123258	0.042854949
ENSCPOG00000027490	MDM1	4.169416706	9.769661319	0.005157633
ENSCPOG00000008084	FAM54A	4.125547739	3.856703483	0.030565342
ENSCPOG00000000952	PDZD7	4.11635366	6.512337806	0.011388281
ENSCPOG00000012483	DPY19L3	4.082426545	3.685306304	0.033191016
ENSCPOG00000005080	SLITRK3	4.065125319	4.945205083	0.019271366
ENSCPOG00000004028	PIGA	4.042726709	6.880778264	0.010237523
ENSCPOG00000003560	STIL	3.93716968	3.026036993	0.04702684
ENSCPOG00000022891	PDZD3	3.934295569	2.951389417	0.049092351
ENSCPOG00000024787	CD72	3.915495636	4.160762985	0.026598169
ENSCPOG00000004589	PPM1E	3.910330114	4.380734956	0.024179763
ENSCPOG00000000531	ZNF438	3.904757643	3.141012177	0.044080434
ENSCPOG00000001446	FANCM	3.897084433	5.686128025	0.014782163
ENSCPOG00000020336	PNPLA3	3.827444027	3.98953658	0.028732973
ENSCPOG00000014676	SKIDA1	3.82301271	6.668186954	0.01087921
ENSCPOG00000006079	CA14	3.805309166	6.157709683	0.012686825

ENSCPOG00000002635	MBOAT2	3.798945704	4.999708136	0.018876856
ENSCPOG00000010542	PGBD5	3.716821689	3.41076165	0.038128719
ENSCPOG00000005984	AHRR	3.572370439	4.849385561	0.01999493
ENSCPOG00000008233	BAI1	3.548914673	3.035013423	0.046786844
ENSCPOG00000012474	HAS2	3.51855335	7.900874694	0.007822293
ENSCPOG00000004564	HENMT1	3.296395628	4.212958772	0.025993365

Supplementary Table 4: genes expressed in late pregnancy but not in mid pregnancy. Expression in mid pregnancy is higher than 3tmp and significant at the 5% level based on one sample one tailed t-test on log_10 transformed data.

Ensembl Gene ID	Associated Gene Name	LT-AV [tpm]	T-VALUE	P-VALUE
ENSCPOG00000003207	VSIG8	37.97219604	3.580374835	0.013219023
ENSCPOG00000022312	SPC24	22.28869824	11.04642608	0.000211631
ENSCPOG00000006079	CA14	18.75328943	3.579637263	0.013227853
ENSCPOG00000005926	SPC25	18.48704256	17.27338634	3.64219E-05
ENSCPOG00000003286	AURKB	17.11575788	13.10983711	0.000108179
ENSCPOG00000015373	FOXM1	16.72319823	17.07448973	3.8131E-05
ENSCPOG00000007305	CEP55	16.44403948	22.27039918	1.32865E-05
ENSCPOG00000015574	TYMS	16.19975973	9.238129647	0.000423855
ENSCPOG00000003918	ADAMTS8	14.12521805	3.182053954	0.01920138
ENSCPOG00000014147	MKI67	13.45266179	16.17724575	4.7211E-05
ENSCPOG00000001228	CDCA8	13.01712435	9.603294732	0.000364818
ENSCPOG00000009002	BUB1B	12.7625169	12.57945515	0.000127234
ENSCPOG00000008312	KIF20A	12.75663419	10.35963713	0.000271792
ENSCPOG00000001241	ZWILCH	11.52450395	15.05642621	6.26982E-05
ENSCPOG00000004203	CDKN3	11.40293944	7.772817827	0.000822536
ENSCPOG00000004318	KIF23	11.08093699	13.91076908	8.56633E-05
ENSCPOG00000010632	KIFC1	11.07855026	8.597952681	0.000559023
ENSCPOG00000003499	MARCO	11.0216194	4.198275205	0.007775931
ENSCPOG00000004807	DLGAP5	10.9667526	10.17192118	0.000291826
ENSCPOG00000014591	MELK	10.69539358	14.33177247	7.61672E-05
ENSCPOG00000008456	PKMYT1	10.25342757	6.067598061	0.002086663
ENSCPOG00000015019	NUF2	10.11530022	11.34375778	0.000190773
ENSCPOG00000004304	POLE2	9.953873987	7.966387863	0.000748855
ENSCPOG00000004744	ECT2	9.850202307	14.87971807	6.56891E-05

ENSCPOG00000001220	NCAPG	9.844134441	8.848361901	0.000500553
ENSCPOG00000026600	ANKRD37	9.756068964	2.943123974	0.024323021
ENSCPOG00000024081	OIP5	9.7163386	15.14409577	6.12774E-05
ENSCPOG00000010279	UBE2T	9.614338428	7.279577931	0.00105513
ENSCPOG00000008318	SGOL1	9.577060767	9.766014742	0.000341816
ENSCPOG00000005838	NDC80	9.53013149	8.546854367	0.000571977
ENSCPOG00000000820	CENPP	9.418740784	9.126275412	0.000444275
ENSCPOG00000014105	RAD51	9.052650676	7.850848434	0.0007918
ENSCPOG00000027258	MIS18A	8.871154412	8.33486459	0.000629863
ENSCPOG00000013158	BUB1	8.704824398	9.126536037	0.000444226
ENSCPOG00000006754	CDCA5	8.600540546	12.53511354	0.000129009
ENSCPOG00000000878	HMMR	8.38349859	10.74117382	0.000236079
ENSCPOG00000014400	TGM1	8.221187407	9.232546849	0.000424846
ENSCPOG00000002161	KIF2C	8.12879627	10.21654499	0.000286902
ENSCPOG00000004015	CENPN	8.017349444	7.212559566	0.00109271
ENSCPOG00000020458	MYBL2	8.005786023	4.97041589	0.004309676
ENSCPOG00000006055	GTSE1	8.003044291	5.907418847	0.002303237
ENSCPOG00000026951	FAM83D	7.960570394	9.536482184	0.000374817
ENSCPOG00000004836	CCNE1	7.886250451	7.325543901	0.001030276
ENSCPOG00000011054	DYNC1I1	7.873065686	4.419703468	0.006514693
ENSCPOG00000002934	SPAG5	7.857624798	13.09517445	0.000108656
ENSCPOG00000004172	CCDC148	7.810910578	4.058335936	0.008726218
ENSCPOG00000010155	NCAPH	7.668629502	5.980749981	0.00220082
ENSCPOG00000025118	CENPA	7.58884668	5.694649532	0.002635381
ENSCPOG00000008700	IL1RN	7.497515203	3.66381986	0.012263842
ENSCPOG00000014099	CASC5	7.45032837	12.60052253	0.000126401
ENSCPOG00000006977	CENPK	7.363234184	6.392238537	0.001719533
ENSCPOG00000004994	HJURP	7.243088089	8.681603649	0.000538592

ENSCPOG00000013206	CENPM	7.185528667	5.948012107	0.002245836
ENSCPOG00000001515	ZNF367	7.028161392	4.270159878	0.007336476
ENSCPOG00000013281	SKA1	6.916919856	6.821300082	0.001348413
ENSCPOG00000014264	SPINK5	6.916887872	3.700143976	0.011873914
ENSCPOG00000004603	ASPM	6.761911233	10.39026253	0.000268688
ENSCPOG00000002037	ARHGAP11A	6.67472445	6.144552945	0.001991539
ENSCPOG00000003710	EZH2	6.624267028	5.890155124	0.002328194
ENSCPOG00000022571	PSMC3IP	6.492961904	2.864948394	0.026333661
ENSCPOG00000010305	CDC6	6.479527727	3.118090844	0.020437028
ENSCPOG00000005221	CHI3L1	6.468882314	4.770334653	0.004986571
ENSCPOG00000023425	SHCBP1	6.457332238	6.016843212	0.002152468
ENSCPOG00000011264	NEK2	6.449727555	6.852560569	0.001325437
ENSCPOG00000003867	ADAM12	6.449433644	4.581807981	0.005746378
ENSCPOG00000003302	KIF20B	6.421164671	8.046246697	0.00072085
ENSCPOG00000008154	KRT78	6.394482672	10.47786185	0.000260049
ENSCPOG00000026992	TMPRSS11B	6.259558285	2.357375026	0.04517487
ENSCPOG00000009389	NEU2	6.173086341	4.755333009	0.005042369
ENSCPOG00000010100	CHTF18	6.109990738	3.70828109	0.011788606
ENSCPOG00000014374	CENPF	5.992395661	5.934995281	0.002264049
ENSCPOG00000011049	PTGES	5.916090482	19.92372181	2.06795E-05
ENSCPOG00000022514	CCSAP	5.681817135	6.128803118	0.002010566
ENSCPOG00000005492	KIF15	5.540002268	6.05987542	0.002096514
ENSCPOG00000027174	KIF18A	5.367800584	8.166510686	0.000681079
ENSCPOG00000007050	NPL	5.254672407	3.515771326	0.014019836
ENSCPOG00000021476	TRPV4	5.092645882	5.125665103	0.003860505
ENSCPOG00000002270	CDC45	5.092047722	2.397086708	0.043245469
ENSCPOG00000008286	DBF4	5.076675364	7.35361167	0.001015456
ENSCPOG00000004384	DCK	5.069708165	2.804663819	0.028016345

ENSCPOG00000013839	DEPDC1	4.910951995	4.642153071	0.005488753
ENSCPOG00000005361	IQGAP3	4.812570382	3.108864366	0.020622867
ENSCPOG00000000693	SASS6	4.808459919	5.225950616	0.003600593
ENSCPOG00000010952	B4GALNT3	4.772081487	2.64347967	0.033159369
ENSCPOG00000004081	TRIP13	4.630369186	3.599903498	0.012987754
ENSCPOG00000008153	HSD17B14	4.500661976	3.75086551	0.011353931
ENSCPOG00000014965	DTL	4.433920415	2.879973425	0.025932683
ENSCPOG00000001013	CENPT	4.377782564	2.796021208	0.028267628
ENSCPOG00000007425	DIAPH3	4.338191225	8.481553058	0.000589077
ENSCPOG00000013130	PRR11	4.322316594	3.685614913	0.012028075
ENSCPOG00000006691	GPR85	4.31705563	10.42227207	0.00026549
ENSCPOG00000014959	CCNF	4.293380777	4.174491349	0.007928285
ENSCPOG00000003478	TRAIP	4.283718409	2.611844891	0.034291438
ENSCPOG00000003950	C5AR_CAVPO	4.268758354	4.210673303	0.007697914
ENSCPOG00000013876	FCN1	4.227679495	3.345903986	0.016416732
ENSCPOG00000022108	FAM89A	4.209132685	2.547505559	0.036732399
ENSCPOG00000002722	SGOL2	4.045207391	2.588445156	0.035157194
ENSCPOG00000013781	EPGN	3.987691819	3.366117692	0.016107356
ENSCPOG00000023563	KLHDC7A	3.982529713	2.666767158	0.032353271
ENSCPOG00000003560	STIL	3.825688375	2.870789412	0.026176932
ENSCPOG00000007680	FBXO5	3.679293906	2.362745644	0.044908402
ENSCPOG00000010816	CCDC99	3.598772572	2.744585477	0.029817999
ENSCPOG00000010076	ABCA7	3.378690834	2.45162766	0.04074425

Supplementary Table 5: genes turned off in late pregnancy but expressed in mid pregnancy.

Ensembl Gene ID	Associated Gene Name	MT-AV	T VAL MT	P VAL MT
ENSCPOG00000011280	MGAM	34.37864585	30.20836077	0.000547019
ENSCPOG00000022112	SCXB	26.62829059	24.19320732	0.000852063
ENSCPOG00000011524	COL9A2	19.22503355	5.444792997	0.016057751
ENSCPOG00000010149	CSPG5	17.93392385	7.817859936	0.007985319
ENSCPOG00000020028	HS3ST5	14.72672374	10.65709286	0.004345127
ENSCPOG00000011277	CACNA1D	14.1205194	16.96491185	0.001728266
ENSCPOG00000025187	NPW	13.19880852	12.47722197	0.003181077
ENSCPOG00000006519	LECT1	12.96579024	6.945537816	0.010053198
ENSCPOG00000000541	DEFB136	12.19513489	5.428718062	0.016148424
ENSCPOG00000015654	LSAMP	12.13555213	9.206989584	0.005796044
ENSCPOG00000019658	SNORD48	11.49234471	6.404662435	0.011760901
ENSCPOG00000017796	snosnR60_Z15	11.27960481	4.364949263	0.024342382
ENSCPOG00000018562	SNORA4	11.06732928	2.993183595	0.047920344
ENSCPOG00000024199	HIF3A	10.75212133	16.31264666	0.00186845
ENSCPOG00000018401	SNORA33	10.62508728	3.042589933	0.046585634
ENSCPOG00000011390	TRPM3	10.41794189	11.31576775	0.003859673
ENSCPOG00000024127	PRKCG	10.23775518	4.303634221	0.024989439
ENSCPOG00000019975	CNR1	9.939697355	5.322603805	0.016766379
ENSCPOG00000010052	TCAP	9.774200406	17.89925985	0.001553361
ENSCPOG00000009157	RXFP2	9.462985428	3.433407924	0.037682636
ENSCPOG00000020301	SNORD12	9.349367918	8.459053621	0.006844426
ENSCPOG00000018242	SNORD74	9.09758675	3.152784826	0.043793757
ENSCPOG00000000329	MPPED2	8.986978101	3.169119465	0.043400391
ENSCPOG00000029300	SNORD38	8.717709384	6.393998652	0.011798784

ENSCPOG00000023518	SNORD33	8.700951742	8.839380356	0.006278923
ENSCPOG00000018365	SNORA52	8.64483505	13.56441245	0.002695535
ENSCPOG00000006429	FGF10	8.642036394	6.095085619	0.012938782
ENSCPOG00000001880	MTCP1	8.315845102	2.96011037	0.048844452
ENSCPOG00000010139	OLAH	8.077416267	3.094789682	0.04523235
ENSCPOG00000008473	VAMP1	7.98285838	4.052247235	0.027923063
ENSCPOG00000020219	MEX3A	7.926489166	6.063048834	0.013070549
ENSCPOG00000012191	SPHK1	7.839506498	4.618913916	0.021907565
ENSCPOG00000005471	IGF2BP2	7.832173946	3.260903845	0.041281495
ENSCPOG00000010546	HUNK	7.717972224	5.864668569	0.013932516
ENSCPOG00000022690	TOP1MT	7.64123455	8.075670124	0.007494828
ENSCPOG00000007686	LYPD6	7.615074331	15.53487524	0.002059042
ENSCPOG00000014250	ATP1B2	7.514779203	6.563766199	0.011216457
ENSCPOG00000003558	TAL1	7.48139324	3.566390823	0.035209045
ENSCPOG00000027324	SNORD33	7.42633808	6.87391201	0.01025736
ENSCPOG00000003979	CLNK	7.395577043	4.017772686	0.028364125
ENSCPOG00000010995	CATSPERG	7.390725688	3.043780531	0.046554127
ENSCPOG00000021594	COL23A1	7.360528688	28.17856571	0.000628511
ENSCPOG00000010865	SLC9A5	7.354689161	3.409941418	0.038145018
ENSCPOG00000012010	ITGBL1	7.022704483	11.73122598	0.003594023
ENSCPOG00000002756	ITGA10	7.016525502	35.70008694	0.000391851
ENSCPOG00000002686	SLC26A7	6.974492714	4.632797321	0.021784901
ENSCPOG00000029326	snoU13	6.938592802	7.251500254	0.009245632
ENSCPOG00000013112	FAM46C	6.736438821	5.984696792	0.013401303
ENSCPOG00000009453	ZNF558	6.734968665	9.849527526	0.005075594
ENSCPOG00000023622	FAM43B	6.646880224	8.820748591	0.006304977
ENSCPOG00000001651	MYOT	6.605670951	5.53342909	0.015571023
ENSCPOG00000020541	GNG7	6.494052268	5.745282269	0.014492369

ENSCPOG00000012005	TESC	6.344180061	34.75977403	0.000413311
ENSCPOG00000004668	GPRASP2	6.326934287	3.911637174	0.029787518
ENSCPOG00000014097	AMIGO1	6.318888233	4.595464757	0.022117055
ENSCPOG00000014734	AIF1L	6.253319769	3.142597484	0.044041674
ENSCPOG00000007504	CDH8	6.235479232	7.916259044	0.007792625
ENSCPOG00000008237	POU2F1	6.217851259	19.76202695	0.00127539
ENSCPOG00000002979	SYNGAP1	6.216453209	5.374997399	0.016456988
ENSCPOG00000026303	SNORA44	6.20607386	5.441251367	0.016077663
ENSCPOG00000021708	ZNF487P	6.190203994	4.788944118	0.020472056
ENSCPOG00000012135	Nptx2	6.132563632	6.805614871	0.01045782
ENSCPOG00000002682	ARG2	6.094524984	3.915597173	0.029732563
ENSCPOG00000012724	RTKN	6.047360681	6.09913244	0.012922277
ENSCPOG00000011580	SEMA3D	6.036895676	7.887683539	0.007847865
ENSCPOG00000008313	REC8	6.027555361	2.939785259	0.04942499
ENSCPOG00000008733	DNAJC6	5.997721442	8.372769787	0.006983264
ENSCPOG00000011456	DDX25	5.921183445	4.950903285	0.019229556
ENSCPOG00000007626	TMEM182	5.912507077	14.03368284	0.002519615
ENSCPOG00000015034	BMX	5.87110737	21.88909216	0.001040297
ENSCPOG00000029707	SNORA2	5.858803559	6.412260846	0.011734017
ENSCPOG00000001859	PARP6	5.701856758	19.25056943	0.001343783
ENSCPOG00000003205	PEAR1	5.644278602	42.15289121	0.000281157
ENSCPOG00000010731	TAF1C	5.623400852	5.383120344	0.016409776
ENSCPOG00000002509	CIITA	5.60687823	9.174589883	0.005836338
ENSCPOG00000010514	CARNS1	5.584740464	3.841112485	0.030791476
ENSCPOG00000001961	ZNF575	5.584305954	5.957112449	0.013520699
ENSCPOG00000013498	HOXA3	5.563647318	4.605120985	0.022030434
ENSCPOG00000001537	CNTFR	5.540928854	6.726188765	0.010698343
ENSCPOG00000012705	SLC4A5	5.528130194	3.485764588	0.036679704

ENSCPOG00000007999	FAM65B	5.519077144	3.819924401	0.031102706
ENSCPOG00000021876	CCDC62	5.451984515	9.619808713	0.005316997
ENSCPOG00000012831	FRAS1	5.383447135	22.21203969	0.001010358
ENSCPOG00000006861	PTGDR	5.356570294	2.923231169	0.049905094
ENSCPOG00000005315	KLF15	5.328633532	4.980059122	0.019017701
ENSCPOG00000002021	EPHB1	5.292442134	4.11869717	0.027100655
ENSCPOG00000006158	COL8A2	5.227725512	14.11297844	0.002491592
ENSCPOG00000010990	KRBA1	5.226779956	3.259031409	0.041323224
ENSCPOG00000019450	ARHGAP33	5.219416917	3.007146726	0.047537662
ENSCPOG00000024720	SH3D21	5.206626998	3.581337548	0.034945795
ENSCPOG00000013143	KAL1	5.203983626	9.115734197	0.00591061
ENSCPOG00000015533	DOCK5	5.159270734	9.22869286	0.005769284
ENSCPOG00000009497	GLIS1	5.147850327	12.83532661	0.003007626
ENSCPOG00000002707	SFMBT2	5.099959082	6.633891799	0.010988295
ENSCPOG00000006087	DGKH	5.022621997	11.33127327	0.003849239
ENSCPOG00000014064	PRDM1	5.020943554	3.002603051	0.047661709
ENSCPOG00000014092	ATXN7L2	4.999737103	6.492069696	0.01145707
ENSCPOG00000004661	HEPACAM2	4.993939644	4.105445406	0.027261819
ENSCPOG00000004316	SEMA3G	4.978657198	4.168143949	0.026511406
ENSCPOG00000010363	SLC10A6	4.977819327	10.60663447	0.004386023
ENSCPOG00000023279	TPPP2	4.967517772	4.302037255	0.025006626
ENSCPOG00000006542	LETM2	4.915853073	4.304386661	0.024981347
ENSCPOG00000019833	KIF26A	4.902746979	6.613213099	0.011054855
ENSCPOG00000007401	SDK2	4.869163451	4.534139089	0.022679013
ENSCPOG00000003908	CARD11	4.818410219	4.711804064	0.021105669
ENSCPOG00000009484	ERCC6	4.781408689	4.815796876	0.020258038
ENSCPOG00000026626	WDR86	4.753968394	6.201246965	0.012515918
ENSCPOG00000004956	IRK2_CAVPO	4.72548306	6.301485942	0.012135162

ENSCPOG00000012901	ARID3B	4.701060735	4.021304759	0.028318472
ENSCPOG00000019470	RERGL	4.699566744	4.014514601	0.028406332
ENSCPOG00000001958	PPARGC1B	4.623561673	10.08537243	0.004844384
ENSCPOG00000013780	KIAA1324L	4.544966979	8.863589916	0.006245309
ENSCPOG00000003557	ANKRD16	4.541936403	3.078898105	0.04563833
ENSCPOG00000003792	KBTBD3	4.510175281	3.469495306	0.03698719
ENSCPOG00000011063	FIGN	4.504563907	11.47103111	0.003757063
ENSCPOG00000007596	TNFRSF9	4.458577931	8.457789999	0.00684643
ENSCPOG00000001222	PRELID2	4.430142888	3.981669272	0.028836987
ENSCPOG00000005029	NUAK1	4.428880215	4.251536264	0.025559176
ENSCPOG00000019676	SLMO1	4.415821725	4.270315668	0.025351639
ENSCPOG00000001406	SCAI	4.400545206	9.115836313	0.00591048
ENSCPOG00000000935	BBS9	4.392125339	3.228148671	0.042020295
ENSCPOG00000011243	B3GNTL1	4.378804899	3.528759109	0.035884659
ENSCPOG00000003564	HRC	4.344747245	10.50088302	0.004473621
ENSCPOG00000001067	TMEM26	4.341018825	3.096029774	0.045200886
ENSCPOG00000011399	FAAH	4.285405374	5.023758484	0.018706542
ENSCPOG00000011602	STXBP4	4.272174434	3.291972691	0.04059784
ENSCPOG00000009749	BZRAP1	4.265985005	3.295563083	0.040519885
ENSCPOG00000005203	MAGEE1	4.252216252	6.384082258	0.011834174
ENSCPOG00000009467	ZNF711	4.234853413	6.588615001	0.011134812
ENSCPOG00000012735	LONRF3	4.230887573	6.74261959	0.010647918
ENSCPOG00000010619	ZNF35	4.185653744	11.52788712	0.003720506
ENSCPOG00000010801	ZFP112	4.162894721	10.58033852	0.004407564
ENSCPOG00000003316	NRXN1	4.149429889	3.006321741	0.047560151
ENSCPOG00000007269	XRRA1	4.139668803	3.560969749	0.035305232
ENSCPOG00000020150	TMEM178B	4.123368664	3.252144067	0.041477237
ENSCPOG00000014189	DYNC2H1	4.110350107	4.660670749	0.021541655

ENSCPOG00000004985	MANEAL	4.075924921	3.81645319	0.031154129
ENSCPOG00000008827	ALS2CR8	4.075831224	5.340081109	0.016662227
ENSCPOG00000011462	IL17D	4.062094988	6.445222169	0.011618447
ENSCPOG00000021122	ZBTB42	4.01794575	4.772383305	0.020605707
ENSCPOG00000012693	ZFHX4	4.01334986	3.573888991	0.035076626
ENSCPOG00000003047	PCDH18	3.984218444	8.387109086	0.006959902
ENSCPOG00000018909	GRIK5	3.982226595	4.705110634	0.021162
ENSCPOG00000011482	MTMR7	3.9585898	3.83676985	0.030854895
ENSCPOG00000006621	USP51	3.949286982	4.973769349	0.019063113
ENSCPOG00000008280	ESRRG	3.929286917	11.98175694	0.003446831
ENSCPOG00000014433	SYT14	3.928713893	5.974693513	0.013444421
ENSCPOG00000002997	CYP4X1	3.900628331	9.863040991	0.005061905
ENSCPOG00000020248	KIAA1383	3.890902511	3.524086674	0.035969849
ENSCPOG00000013001	PLCB2	3.889677827	3.604835291	0.034537659
ENSCPOG00000001518	PPARGC1A	3.882643392	4.641614914	0.021707518
ENSCPOG00000018797	SNORD60	3.822497481	4.543710473	0.022589937
ENSCPOG00000010494	KLRG1	3.808041199	7.350784624	0.009004224
ENSCPOG00000011750	CYP2U1	3.757556416	3.884925255	0.030162102
ENSCPOG00000023974	ZCCHC3	3.728546306	3.00894277	0.047488755
ENSCPOG00000001644	ATP7B	3.707571473	9.903607602	0.005021143
ENSCPOG00000001687	PCDHGB2	3.659350645	4.757930889	0.020723391
ENSCPOG00000014004	OPN3	3.581140557	3.803072896	0.031353507
ENSCPOG00000012883	PRDM5	3.55531152	5.769127237	0.014377924
ENSCPOG00000024962	NAPB	3.470274853	4.1342148	0.026913694
ENSCPOG00000001229	BBS7	3.451498506	6.72328741	0.010707284
ENSCPOG00000005172	NHSL2	3.442740893	3.440145733	0.037551367
ENSCPOG00000002485	KIF27	3.442053572	3.004597362	0.047607206
ENSCPOG00000020913	SNORD35	3.428784286	3.037193647	0.046728817

ENSCPOG00000004119	PLEKHM3	3.416143049	2.929800301	0.049713785
ENSCPOG00000002661	RTTN	3.410734075	11.38888026	0.00381084
ENSCPOG00000010468	ZNF618	3.361685849	2.992486773	0.047939556
ENSCPOG00000003530	GLT25D2	3.335607063	3.630096442	0.034106541
ENSCPOG00000001158	PAG1	3.291018235	2.957183915	0.04892744
ENSCPOG00000005997	TSKS	3.264325833	6.329142766	0.012033127

Supplementary Table 6: comparison of genes different between ripe and unripe human cervix according to (Hassan et al., 2009) and the fold change between mid pregnancy and late pregnancy in the guinea pig cervix.

**AGREE**	Human fold change	FDR	Guinea pig fold change	p-vlaue	REMARK
ESR1	0.85	5.00E-03	0.65	4.96E-03	estrogen receptor 1
BRM1	0.88	2.66E-02	0.73	2.52E-03	Polypromo 1
SIGLEC1	1.41	3.22E-02	1.89	9.48E-03	Sialoadhaesine
CALD1	1.44	7.00E-04	1.69	1.27E-02	Caldesmon 1
TPM1	1.66	2.90E-03	1.53	2.45E-02	Tropomyosin 1
IGFBP4	1.84	3.71E-02	2.75	2.18E-03	Insulin-like growth factor binding protein 4
PLN	1.95	1.30E-03	2.03	3.97E-02	Phosopholamban
COL4A2	1.99	5.00E-03	1.37	4.64E-02	Basal lamina but laminin is downregulated in gp!
VCAN	2.82	1.00E-05	1.61	1.95E-02	Versican
**DISAGEE**					
TACC2	0.85	3.20E-02	1.36	6.08E-02	Transforming, acidic coiled-coil containing protein 2
NAP1L1	1.22	1.00E-	0.84	4.10E-	Nucleosome assembly protein 1-like 1

		04		02	
PDE1A	1.31	1.90E-03	0.39	2.27E-03	Phosphodiesterase 1A, calmodulin-dependent
MEF2C	1.32	3.36E-02	0.62	3.89E-02	Myocyte enhancer factor 2C
CCDC50	1.44	2.90E-03	0.75	5.60E-03	Coiled-coil domain containing 50
TCF4	1.45	1.00E-04	0.34	3.18E-04	transcription factor 4
BNC2	1.45	3.00E-04	0.45	3.06E-04	Basonuclin 2, transcription factor
BGN	1.45	5.00E-03	0.62	2.78E-02	biglycan
PTPRG	1.46	4.53E-02	0.75	3.24E-05	Protein tyrosine phosphatase, receptor type, G
NR2F2	1.49	3.36E-02	0.54	7.94E-04	Nuclear receptor subfamily 2, group F, member 2
PLXND1	1.49	4.54E-02	0.78	4.75E-02	Plexin D1, highly expressed in cervical cancer antiangiogenic
ADCY3	1.50	3.63E-02	0.84	1.60E-02	Adenylate cyclase 3
CYYR1	1.51	3.02E-02	0.56	1.35E-02	Cysteine/tyrosine-rich 1
FN1	1.52	4.40E-03	0.73	5.52E-02	Fibronectin 1
ADAMTS2	1.53	1.30E-03	0.80	2.67E-02	ADAM metallopeptidase
LAMC1	1.57	2.55E-	0.82	3.37E-	Laminin gamma 1

		02		02	
SYT11	1.68	8.10E-03	0.60	2.35E-03	Synaptotagmin XI
EFEMP2	1.68	1.45E-02	0.83	4.64E-02	EGF-containing fibulin-like extracellular matrix protein 2
LAMA2	1.73	3.25E-02	0.71	2.41E-03	Laminin alpha 2
FOXO1	1.78	5.00E-03	0.71	4.22E-02	Forkhead box O1
ICAM2	1.82	2.04E-02	0.54	5.40E-02	Intercellular adhesion molecule 2
RHOQ	1.88	1.00E-05	0.76	5.88E-02	ras homolog gene family, member Q
SPOCD1	1.92	8.80E-03	0.68	4.62E-02	SPOC domain containing 1
TIMP3	2.01	1.00E-04	0.51	1.31E-02	TIMP metallopeptidase inhibitor 3
PXDN	2.18	2.05E-02	0.63	5.84E-04	Peroxidasin homolog
FCRL5	2.28	3.36E-02	0.20	2.24E-02	Fc receptor-like 5

Supplementary Figure 1: comparison of log_2 fold difference between ripe and unripe human cervix compared to log_2 fold change in guinea pig cervix between mid and late pregnancy. Included are data from 79 genes that are statistically significantly different between human unripe and ripe cervix and for which the corresponding gene has been measured in the guinea pig cervix. The correlation is low, 0.054. This result suggests limited similarity in cervical gene regulation in humans and guinea pigs. For a discussion of the limitations of this comparison see the main text.

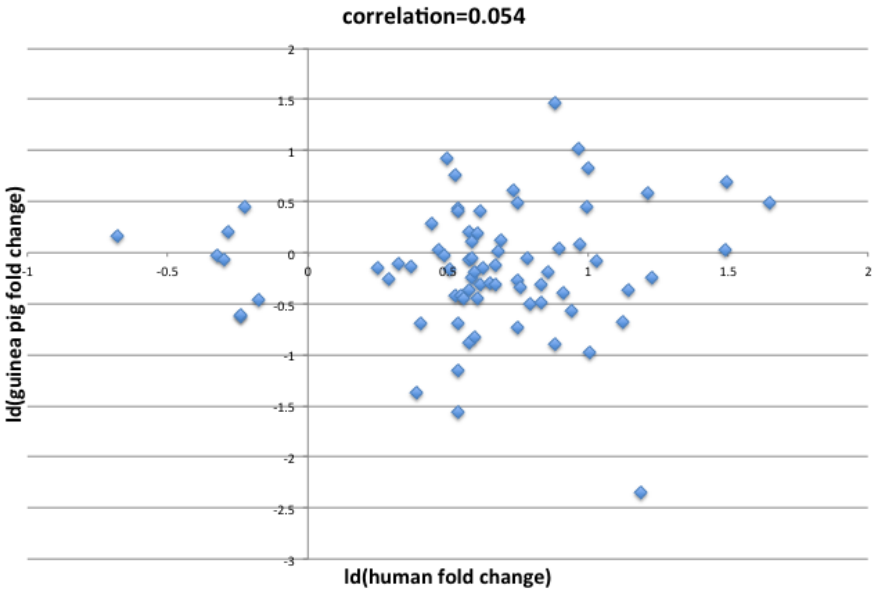

Suppl Figure 1
